# Supplementary material for: Systemic Immune Inflammation Index (SII), System Inflammation Response Index (SIRI) and Risk of All-Cause Mortality and Cardiovascular Mortality: A 20-Year Follow-Up Cohort Study of 42,875 US Adults
Source: J Clin Med. 2023 Jan 31;12(3):1128. doi: 10.3390/jcm12031128 (PMC9918056; doi:10.3390/jcm12031128)
Supplement: Supplementary file 1 [file jcm-12-01128-s001.zip › jcm-2160802-supplementary.pdf]

Table S1. Baseline characteristics of the general adult population included in NHANES 1999–2018.

Variables	Total (*n* = 42,875)
Age, years	44 (18)
Male, %	4010 (47.8)
Race/ethnicity, %	
Mexican American	8578 (20.0)
Other Hispanic	3727 (8.7)
Non-Hispanic White	17,029 (39.7)
Non-Hispanic Black	9287 (21.7)
Other race	4254 (9.9)
Education level, %	
Below high school	11,715 (27.3)
High school	9633 (22.5)
Above high school	21,527 (50.2)
Poverty, %	9243 (21.6)
Smoking status, %	
Never smokers	24,908 (58.1)
Former smokers	8696 (20.3)
Current smokers	9271 (21.6)
Drinking status, %	
Nondrinkers	9554 (22.3)
Low-to-moderate drinkers	30,005 (70.0)
Heavy drinkers	3316 (7.7)
BMI status, %	
<25.0	13,872 (32.4)
25.0–29.9	14,087 (32.9)
>29.9	14,916 (34.8)
Physical activity, %	
Inactive	10,511 (24.5)
Insufficiently active	16,057 (37.5)
Active	16,307 (38.0)
Energy intake, kcal/day	1961.00 [1475.00, 2574.18]
eGFR, ml/min/1.73 m^2	100.05 (23.28)
ALT, U/L	21.00 [16.00, 28.00]
AST, U/L	22.00 [19.00, 27.00]
Hypertension, %	11,594 (27.0)
Diabetes, %	3776 (8.8)
WBC count, 10^3/μL	6.90 [5.70, 8.40]
Neutrophils count, 10^3/μL	4.00 [3.10, 5.10]
Monocyte count, 10^3/μL	0.50 [0.40, 0.60]
Lymphocyte count, 10^3/μL	2.10 [1.70, 2.60]
Platelet count, 10^3/μL	248.00 [211.00, 293.00]
SII	468.84 [335.37, 655.56]
SIRI	0.99 [0.68, 1.43]

Normally distributed continuous variables are described as means and SD, and continuous variables without a normal distribution are presented as medians [interquartile ranges]. Categorical variables are presented as numbers (percentages). eGFR, estimated glomerular filtration rate; ALT, alanine aminotransferase; AST, aspartate aminotransferase; WBC, white blood cell; SII, systemic immune-inflammatory index; SIRI, systemic inflammatory response index.

Table S2. Hazard ratios of cardiovascular diseases and all-cause mortality according to quantiles of SII and SIRI stratified by gender.

	Cardiovascular mortality		All-cause mortality	
	**Female (*n* = 21,792)**	**Male (*n* = 21,083)**	**Female (*n* = 21,792)**	**Male (*n* = 21,083)**
**SII**				
Q1 (<335.36)	Reference	Reference	Reference	Reference
Q2 (355.36–468.83)	1.03 (0.78–1.37)	1.13 (0.87–1.47)	0.97 (0.84–1.12)	1.01 (0.89–1.14)
Q3 (468.84–655.55)	1.12 (0.85–1.49)	1.21 (0.93–1.57)	1.03 (0.89–1.18)	1.04 (0.91–1.17)
Q4 (>655.56)	1.38 (1.06–1.81)	1.30 (1.01–1.67)	1.33 (1.17–1.52)	1.26 (1.12–1.42)
p for trend	0.047	0.227	<0.001	<0.001
p for interaction	0.756		0.722	
**SIRI**				
Q1 (<0.68)	Reference	Reference	Reference	Reference
Q2 (0.68–0.98)	0.84 (0.64–1.12)	0.93 (0.67–1.28)	0.96 (0.84–1.10)	1.02 (0.88–1.19)
Q3 (0.99–1.42)	1.08 (0.82–1.42)	1.13 (0.84–1.53)	1.13 (0.99–1.30)	1.18 (1.03–1.36)
Q4 (>1.43)	1.32 (1.01–1.72)	1.46 (1.10–1.94)	1.40 (1.23–1.60)	1.38 (1.21–1.58)
p for trend	0.008	0.002	<0.001	<0.001
p for interaction	0.907		0.152	

Data are presented as HR (95% CI) unless indicated otherwise. The multiple cox analysis was conducted by quantiles of SII or SIRI adjusted for age (continuous), race/ethnicity (Mexican American, Other Hispanic, Non-Hispanic White, Non-Hispanic Black or Other), education level (below high school, high school, or above high school), family poverty income ratio (<1.0, or ≥1.0), drinking status (nondrinkers, low-to-moderate drinkers, or heavy drinkers), smoking status (never smokers, former smokers, or current smokers), BMI (<25.0, 25.0–29.9, or >29.9), physical activity (inactive, insufficiently active, or active), total energy intakes (in quartiles), estimated glomerular filtration rate (continuous), alanine aminotransferase (in quartiles), aspartate aminotransferase (in quartiles), self-reported hypertension (yes or no), and self-reported diabetes (yes or no). CI, confidence interval; HR, hazard ratio; SII, systemic immune-inflammation index; SIRI, systemic inflammation response index.

Table S3. Hazard ratios of cardiovascular diseases and all-cause mortality according to quantiles of SII and SIRI stratified age.

	Cardiovascular mortality		All-cause mortality	
	**Age <60 (*n* = 32,474)**	**Age ≥60 (*n* = 10,401)**	**Age <60 (*n* = 32,474)**	**Age ≥60 (*n* = 10,401)**
**SII**				
Q1 (<335.36)	Reference	Reference	Reference	Reference
Q2 (355.36–468.83)	1.01 (0.66–1.55)	1.08 (0.87–1.35)	0.87 (0.73–1.03)	1.03 (0.92–1.15)
Q3 (468.84–655.55)	1.23 (0.82–1.85)	1.14 (0.92–1.41)	0.96 (0.81–1.14)	1.06 (0.95–1.18)
Q4 (>655.56)	1.31 (0.88–1.95)	1.33 (1.08–1.64)	1.06 (0.90–1.25)	1.39 (1.26–1.54)
p for trend	0.430	0.039	0.110	<0.001
p for interaction	0.940		0.008	
**SIRI**				
Q1 (<0.68)	Reference	Reference	Reference	Reference
Q2 (0.68–0.98)	0.75 (0.48–1.17)	0.96 (0.75–1.22)	0.84 (0.70–1.01)	1.11 (0.98–1.25)
Q3 (0.99–1.42)	0.99 (0.66–1.50)	1.18 (0.93–1.49)	1.11 (0.94–1.31)	1.24 (1.10–1.40)
Q4 (>1.43)	1.39 (0.95–2.04)	1.56 (1.24–1.95)	1.12 (0.94–1.32)	1.66 (1.48–1.86)
p for trend	0.022	<0.001	0.005	<0.001
p for interaction	0.528		0.003	

Data are presented as HR (95% CI) unless indicated otherwise. The multiple cox analysis was conducted by quantiles of SII or SIRI adjusted for sex (male or female), race/ethnicity (Mexican American, Other Hispanic, Non-Hispanic White, Non-Hispanic Black or Other), education level (below high school, high school, or above high school), family poverty income ratio (<1.0, or ≥1.0), drinking status (nondrinkers, low-to-moderate drinkers, or heavy drinkers), smoking status (never smokers, former smokers, or current smokers), BMI (<25.0, 25.0–29.9, or >29.9), physical activity (inactive, insufficiently active, or active), total energy intakes (in quartiles), estimated glomerular filtration

rate (continuous), alanine aminotransferase (in quartiles), aspartate aminotransferase (in quartiles), self-reported hypertension (yes or no), and self-reported diabetes (yes or no). CI, confidence interval; HR, hazard ratio; SII, systemic immune-inflammation index; SIRI, systemic inflammation response index.

Table S4. Hazard ratios of cardiovascular diseases and all-cause mortality after excluding events occurred at the first two years of the follow-up.

	Cardiovascular mortality	All-cause mortality
	**HR (95%CI)**	**HR (95%CI)**
SII		
Q1 (<335.25)	Reference	Reference
Q2 (335.25–468.40)	1.10 (0.90–1.35)	1.01 (0.91–1.11)
Q3 (468.41–654.25)	1.18 (0.96–1.44)	1.05 (0.95–1.15)
Q4 (>654.26)	1.33 (1.10–1.62)	1.28 (1.17–1.40)
p for trend	0.027	<0.001
SIRI		
Q1 (<0.67)	Reference	Reference
Q2 (0.67–0.97)	0.89 (0.71–1.11)	1.00 (0.90–1.11)
Q3 (0.98–1.42)	1.13 (0.91–1.39)	1.17 (1.05–1.29)
Q4 (>1.43)	1.40 (1.14–1.71)	1.37 (1.24–1.51)
p for trend	<0.001	<0.001

Data are presented as HR (95% CI) unless indicated otherwise. The multiple cox analysis was conducted by quantiles of SII or SIRI adjusted for age (continuous), sex (male or female), race/ethnicity (Mexican American, Other Hispanic, Non-Hispanic White, Non-Hispanic Black or Other), education level (below high school, high school, or above high school), family poverty income ratio (<1.0, or ≥1.0), drinking status (nondrinkers, low-to-moderate drinkers, or heavy drinkers), smoking status (never smokers, former smokers, or current smokers), BMI (<25.0, 25.0–29.9, or >29.9), physical activity (inactive, insufficiently active, or active), total energy intakes (in quartiles), estimated glomerular filtration rate (continuous), alanine aminotransferase (in quartiles), aspartate aminotransferase (in quartiles), self-reported hypertension (yes or no), and self-reported diabetes (yes or no). CI, confidence interval; HR, hazard ratio; SII, systemic immune-inflammation index; SIRI, systemic inflammation response index.
